# Pupil response components: attention-light interaction in patients with Parinaud’s syndrome

**DOI:** 10.1038/s41598-017-10816-x

**Published:** 2017-08-31

**Authors:** Paola Binda, Torsten Straßer, Krunoslav Stingl, Paul Richter, Tobias Peters, Helmut Wilhelm, Barbara Wilhelm, Carina Kelbsch

**Affiliations:** 1 Department of Translational Research on New Technologies in Medicine and Surgery, University of Pisa, Pisa, Italy; 2 CNR Neuroscience Institute, Pisa, Italy; 3 Pupil Research Group at the Centre for Ophthalmology, University of Tübingen, Tübingen, Germany

## Abstract

Covertly shifting attention to a brighter or darker image (without moving one’s eyes) is sufficient to evoke pupillary constriction or dilation, respectively. One possibility is that this attentional modulation involves the pupillary light response pathway, which pivots around the olivary pretectal nucleus. We investigate this possibility by studying patients with Parinaud’s syndrome, where the normal pupillary light response is strongly impaired due to lesions in the pretectal area. Four patients and nine control participants covertly attended (while maintaining fixation at the center of a monitor screen) to one of two disks located in the left and right periphery: one brighter, the other darker than the background. Patients and control subjects behaved alike, showing smaller pupils when attending to the brighter stimulus (despite no eye movements); consistent results were obtained with a dynamic version of the stimulus. We interpret this as proof of principle that attention to bright or dark stimuli can dynamically modulate pupil size in patients with Parinaud’s syndrome, suggesting that attention acts independently of the pretectal circuit for the pupillary light response and indicating that several components of the pupillary response can be isolated – including one related to the focus of covert attention.

## Introduction

The pupillary light response, or the pupillary constriction evoked by light increments, depends primarily on a dorsal mesencephalic nucleus known as olivary pretectal nucleus^1, 2^. The olivary pretectal nucleus receives input from the retina and sends its output to the pupillomotor center in the Edinger-Westphal nucleus EW^3^, which then controls the activity of the sphincter of the pupil, hence the level of pupillary constriction.

Although light is the primary determinant of pupil size, there are other pupil behaviors with independent neural circuits. The best known of these is the “near response”, the pupillary constriction that accompanies near focus^4, 5^. There are also oscillations of pupil diameter associated with arousal level through noradrenergic signaling^6^; these can either be spontaneous, leading to the so called “pupillary unrest”^7, 8^, or they can be associated with increased attention or memory load, leading to cognitive-related pupil dilation^9, 10^. Finally, there is the transient pupillary constriction observed at the onset of stimuli that do not change luminance, like gratings or isoluminant color modulations^11–13^.

Although the circuits underlying these responses are not completely understood, they all appear to bypass the olivary pretectal nucleus. The best evidence for this hypothesis comes from the study of rare patients with dorsal mesencephalic lesions leading to Parinaud’s syndrome: a selective impairment of the pupillary light response, with normal or quasi-normal near response, onset response and pupillary unrest^14^. While light usually generates a large and brisk constriction of the pupils, it only produces small and sluggish responses in these patients. Based on this and other evidence, it has been suggested that the pupillary light response comprises multiple components^14, 15^: a major component that depends on the pretectal circuit (depleted in Parinaud’s) and a minor component that relies on a different circuit independent of the olivary pretectal nucleus, which could involve a cortical projection to the EW (which bypasses the pretectum) and/or a light-related change of the central sympathetic inhibitory input to the EW.

The recent literature has highlighted yet another type of pupil behavior: a response depending on the interaction between light and attention^16–22^. This is different from the pupillary light response because it is observed with no change of the visual stimulus – the pupils constrict or dilate with a mere shift of covert attention to a brighter or darker stimulus, and despite no eye movement. It is also different from the cognitive-related pupil behavior, because the change of pupil size depends on what is attended to (a bright or a dark stimulus), not on the amount of attention being paid.

These attentional modulations of the pupillary light response are small; however, they are consistent and have been observed in a variety of stimuli and task conditions^20, 23^ and could be reproduced in non-human primates by micro-stimulation of FEF^24^ – a manipulation known to emulate the effects of focused visual spatial attention^25^. Studies are beginning to show how these modulations may prove important not only for the study of pupil behavior, but also as means to probe attentional and visual processes^23^ and even as a practical communication tool: under some conditions, pupil diameter could tell us what people are covertly attending to^20^, a potentially very efficient approach for Brain-Machine Interactions^26^.

With these goals in mind, it is important to elucidate what neural circuits underlie this form of attentional modulation of pupil size. One possibility is that it is implemented through modulatory signals in the standard circuit mediating the pupillary light response, e.g. an attentional enhancement of pretectal activity^16, 27^.

This hypothesis predicts that the attentional modulation of the pupillary light response should be absent in Parinaud’s syndrome patients, where the pupillary light response itself is strongly impaired due to probable pretectal lesion^14^. Here we test this prediction. Although Parinaud’s is a rare syndrome, we recruited four patients, all of which had recently participated in our group’s study with colour pupillography^15^.

## Materials and Methods

Four patients diagnosed with Parinaud’s syndrome participated in the study (see Table 1). They all had a complete ophthalmological examination, including visual acuity, pupil testing, slit lamp examination of the anterior segment and fundus ophthalmoscopy before performing the pupillography. Formal perimetry (Octopus 101) was also performed, one or more times over the years preceding the tests, with stable and unremarkable results. Patients showed no hints for optic nerve atrophy (except a mild temporal optic nerve paleness in patient 2, who nevertheless had an unremarkable 30° static perimetry). However, they all showed impaired pupillary light reactions with preserved near responses (detailed measurements of their pupillary light responses, together with clinical data, are reported in ref. 15). The nine control participants (5 females, mean age ± s.e.: 29.56 ± 1.86 years; maximum 40 years) had normal or corrected to normal vision and were experienced psychophysical participants.

**Table 1:** Patients cohort.

Age (years)	Sex	Diagnosis	Last treatment	Time from last treatment (years)
29	M	Germinoma in the brainstem to thalamus	Radiotherapy	10
26	F	Pinealoblastoma	Radiotherapy	20
41	M	Epidermoid in pinealis	Surgery	26
60	M	Pineal germinoma	Radiotherapy	17

All participants gave their written informed consent; procedures are in line with the Declaration of Helsinki and they were approved by the local ethics committee of the faculty of medicine Eberhardt-Karls-University and of the University Hospital Tübingen.

Visual stimuli were presented on LCD (Liquid Crystal Display) monitor and viewed binocularly, while gaze and pupil size were recorded from the right eye with an infrared camera (see Supplementary Methods for details).

**Figure 1:**
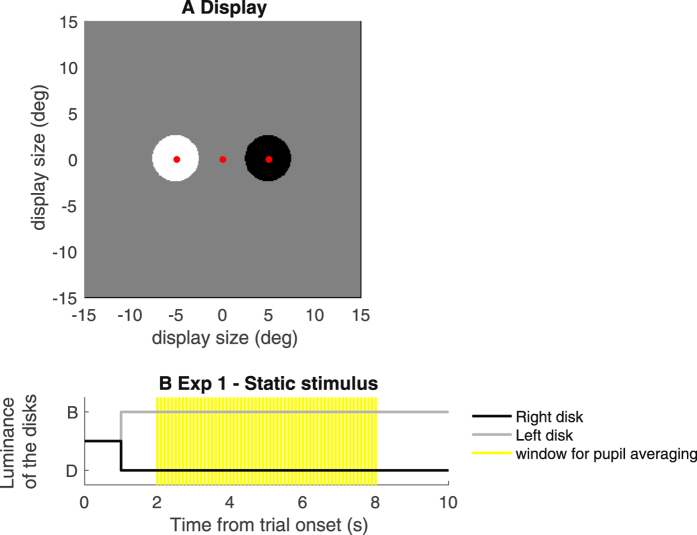
Methods. (**A)** approximately in scale representation of the display screen, with the bright and dark disks shown on the sides of fixation (central red point), and the small dots at the center of each disk. The latter could undergo brief subtle color changes; participants were asked to count these changes, to check that their attention was indeed focused on the left/right stimulus as instructed at the beginning of each block of trials. (**B)** Time-course of luminance for the right and left disks (B and D on the y axis stand for Bright and Dark relative to the background). The disks appeared 1 s into the trial and their luminance remained sustained thereafter; for this reason, we refer to this stimulus as ‘static’ (vs. the dynamic stimulus used for Experiment 2, below). For statistical analyses, eye-tracking data were averaged across the 2–8 s time window (yellow shading).

Trials started with subjects fixating on a 0.5 deg red spot at screen center; after a warning signal, the 1 s pre-stimulus baseline started, followed by presentation of the stimuli. These were two 5 deg diameter disks, centered 5 deg to the left and right of the fixation point, one bright (50 cd/m^2^) the other dark (0.5 cd/m^2^) compared to the 25 cd/m^2^ background against which they appeared (Fig. 1A). Participants were instructed to covertly focus their attention on the left or right stimulus (attention side was indicated at the beginning of a block of trials) while always maintaining their gaze on the fixation point. To clarify this concept, we suggested that they should attend to what they saw “out of the left/right corner of their eyes”. In three/four practice trials, they familiarized with the stimuli and task and we provided feedback on their fixation performance.

The two disks remained visible for 9 seconds, and their disappearance marked the end of each 10 s long trial (Fig. 1B). At the center of each disk was a 0.5 deg green dot that could change color for a short 0.200 s interval. On half the trials one colour change occurred on the attended side (only), on the remaining trials no colour change occurred. Participants were asked to report, vocally at the end of the trial, whether such subtle color change had occurred on the attended side. The positions of the bright and dark disk were pseudo-randomized across trials. Each participant completed a minimum of four blocks (two per attended side) of 8 trials each.

**Figure 2:**
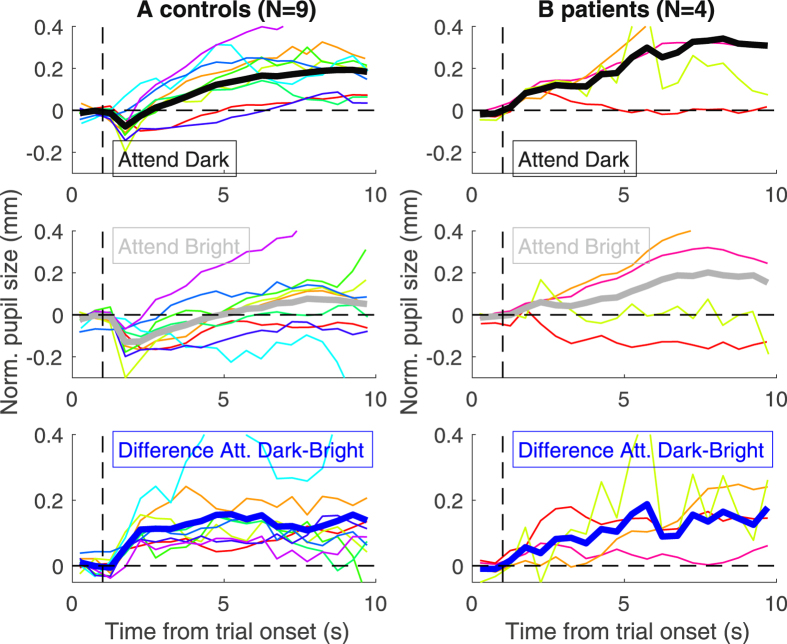
Effect of attention with sustained stimuli. Panels on the left (A) and right (B) report results for the controls and the patients group respectively. Pupil size traces for each trial were binned in 0.5 s contiguous steps, subtracted of the median pupil size in the 1 s pre-stimulus interval (left of the dashed vertical line), then averaged across trials where attention was directed to the dark (top panels) or bright disk (middle panels). The effect of attention is measured as the difference between pupil size traces in the two conditions (bottom panels). Across panels, thin colored lines report single-subject traces and thick continuous lines show the average across participants.

For each trial, we computed the pupil size change relative to the 1 s baseline period preceding the stimulus presentation, then averaged across traces from the same condition (attending bright/dark) yielding the traces in Fig. 2A,B. For the statistical comparisons, we took the median pupil (or gaze position) value in the central 6 seconds of each trial and analyzed these with a linear-mixed model approach (see Supplementary Material for details).

### Data availability

The datasets generated during the current study are available from the corresponding author on reasonable request.

## Results

Procedures essentially reproduced the set up used in previous studies on attentional modulation of pupil size^18^, where participants covertly attended to one of two disks located to the left and right of fixation: brighter and darker than the background.

We ensured that fixation was successfully attained by direct recording of gaze position. We also checked that covert attention was directed according to the instructions by means of a simple change detection task, in which participants had to detect subtle color changes of a dot centered on the attended disk. All control subjects performed above 90% correct. In patients 1–4, percent correct was: 95.8%, 65.6%, 75% and 75% respectively.

In the control subjects (Fig. 2A), we replicated the effect of attention shown in ref. 18: pupil size was smaller when attention was directed to the bright disk than to the dark disk (middle vs. top panel of Fig. 2A), resulting in a pupil size difference between the two attention conditions (bottom panel in Fig. 2A). The effect is similar in the four Parinaud’s syndrome patients (Fig. 2B).

In both subject groups, the effect of attention builds up relatively slowly after stimuli presentation as shown in previous research,^16–18^ and remains approximately constant (and consistently above 0) thereafter. This implies that, although individual pupil traces may have complex shapes (see below), those for the attend dark and attend bright condition run approximately parallel from about 2 s into the trial, displaying a sustained effect of attention.

The statistical reliability of the effect of attention was assessed by means of a linear-mixed model approach, which explicitly represents the variability across-participants as a random effect. The model was entered with the average pupil size values computed, for each trial, in the central 6 seconds of stimulus presentation (yellow shading in Fig. 1B). The effect of attended luminance (bright vs. dark) was significant in both controls (F(1,287) = 22.470, p<0.001) and patients (F(1,157) = 6.878, p<0.01).

While the effect of attention on pupil size is similar across groups (compare the blue trace across Fig. 2A and B), there is a qualitative difference in the shape of individual traces (attending dark or bright; top and middle panels in Fig. 2). For all control participants, there is a clear transient constriction immediately following the onset of the stimuli (Fig. 2A, black and gray traces in the top and middle panels clearly show a dip immediately following the onset of the disks, marked by the vertical dashed line). This effect is noticeably absent in the patients’ traces (Fig. 2B).

We also note that the attentional modulation starts soon after stimulus onset; in the controls, this leads to a larger transient constriction when attending to bright. However, this effect only accounts for a fraction of the effect of attention, which remains significant (F(1,288) = 10.912, p<0.01) after discarding this early modulation by aligning all traces to the peak post-stimulus constriction.

With additional analyses, we checked that gaze shifts towards the attended disk do not explain the effect of attention on pupil size. First, we verified that there was no significant difference of horizontal gaze position (like for pupil size, gaze position was indexed by the median value in the central 6 seconds of the stimulus presentation window) when attention was directed to the left or right (fixed effect of attended side with subject as random effect, F(1,158) = 1.477, p = 0.226 in the patients group and F(1,304) = 1.546, p = 0.215 in the controls group). Second, we confirmed that all findings maintained statistical significance even in a subsample of trials where the median eye position was within ±0.3 deg from screen center (116 in 160 for patients, and 241 in 320 for controls): pupil size reliably modulates with attended luminance in both the patients (F(1,114) = 4.256, p < 0.05) and the controls group (F(1,232) = 15.084, p < 0.001).

Finally, we confirmed the presence of an attentional modulation of pupil size using an alternative paradigm, where the luminance of the attended stimuli swaps periodically (at 0.4 Hz). Three of the patients and six of the controls participated to this supplementary experiment. In both the patients and the controls the effect of attention is seen as a periodic modulation of pupil size at the same frequency and phase as the attended stimulus (Methods and results are presented in the Supplementary Material and shown in Supplementary Figure 1).

## Discussion

Our results in the control participants replicate previous reports that the pupil is sensitive to the interaction between attention luminance: it constricts when attention is covertly directed to a brighter stimulus, despite no change of the luminance intensity or distribution on the retina^18^. Similarly, attending to stimuli that swap their luminance makes the pupil oscillate, in phase with the attended stimulus^20^. We excluded that pupil differences were caused by gaze shifts towards the attended disk, by showing that participants were successful at maintaining fixation: gaze position was not systematically shifted in the direction of attention and average pupil modulations remained the same after excluding any trial with however brief gaze deviation. This concern has been repeatedly addressed in our (and others’) previous work, showing that even substantial (1 deg) gaze deviations towards brighter/darker stimuli fail to account for the pupil modulation during covert attention to the same stimuli^16, 18^, and that pupil differences also occur when covertly attending the brighter or darker two spatially overlapping objects^17^.

Our main finding here is that the effect of attention is observed in patients with Parinaud’s syndrome, who also show relative constriction/dilation when covertly attending the brighter/darker stimulus. Note that we do not claim equal behavior between the two groups, patients and controls, such comparison being inappropriate given the small sample size of the (rare) Parinaud’s patients and given the impossibility to control for possible between group differences, e.g. in terms of age (one of the patients was older than all the controls). Also, patients and controls were not matched by their accuracy on the detection task used to confirm that participants focused attention as directed – the lower accuracy in three out of four patients might simply be due to lack of familiarity with the test settings (in comparison with our controls, who were experienced psychophysical subjects). Together, these factors might have attenuated the effect of attention of pupil size, reducing our chances to detect it in the patients. Yet we did find reliable evidence, in two experiments, that such effect is present in patients with Parinaud’s syndrome. In these patients, a subcortical lesion depletes the main nucleus involved in the pupillary light response – the olivary pretectal nucleus. Thus, the present findings falsify our working hypothesis that the attention modulation of pupillary light responses results from the attentional enhancement of the pretectal response to light. In this way, they further the evidence for the existence of separable components of the pupillary response, as proposed in Wilhelm *et al*.^14^. In particular, they identify a pupillary response that reflects the interaction between light and the focus of attention.

Taken together with prior research^14, 15^, these results suggest that the pupillary light response entails two or more independent components, generated through different pathways: the main pathway through the pretectum, and alternative pathways bypassing the pretectum and modulating activity in the pupillomotor Edinger-Westphal EW nucleus (hence pupil constriction). These alternative pathways may explain the small, sluggish residual light response found in Parinaud’s; they may also integrate information on attention state, explaining the comparable effects of attention we observed in our patients and controls. Current knowledge of the anatomy and physiology of pupillary responses does not allow for outlining the neural circuits that might underlie such alternative pathways. We speculatively outline two general and non-mutually exclusive hypotheses. One possibility is that retinal signals generate a brightness signal in the visual cortex possibly integrating melanopsin signals^28^, which could then activate the EW to generate pupillary constriction. It is easy to incorporate the effect of attention in this model, since focused attention is known to enhance visual stimulus representations in the occipital cortex via feedback from the prefrontal cortex^25^, and electrical stimulation of this very circuit has been recently shown to mimic the attentional modulation of pupillary light responses^24^. Another possibility is that light might down-regulate activity in the sympathetic system, reducing the tone of sympathetic inhibition on EW and producing a net pupillary constriction^14, 15^. Although it is difficult to predict how a change in sympathetic activity would produce a spatially selective modulation of light responses as seen in our paradigm, sympathetic tone is tightly linked to arousal levels, which is modulated by light via melanopsin signals^29^, and constitutes a key factor in attention allocation^30^.

While our experiments focused on revealing the attentional modulation of light responses, pupil traces reveal two other prominent behaviors (top and middle panels of Fig. 2). First, there is progressive pupil dilation over the duration of a trial – in both controls and patients, similarly as in previous reports^18^. We interpret this as the product of cognitive load, or the act of maintaining attention focused on a small spatial region to comply with task instructions. It has been suggested that cognitive-related pupillary dilation depends on noradrenergic signaling^6^, unaffected in Parinaud’s syndrome. This hypothesis is consistent with our observation of similar pupil dilations in our patients and controls; it can also account for the normal “pupil unrest” behavior in Parinaud’s patients^14^, which we confirmed here by means of PST (Pupillographic Sleepiness Test, Supplementary Material).

Second, there is a transient constriction at the onset of the disks. While clearly seen in controls like in ref. 18, this component was absent in the patients. This constriction could reflect the “onset response” typically generated by stimuli that do not alter the overall luminance of the image^12^ – like ours. However, it could also be a transient light response generated by the different time constants^1^ of the fast constriction evoked by luminance increments (the bright disk in our stimulus) and the slower dilation in response to luminance decrements (the dark disk). Since the “onset response” is usually preserved in Parinaud’s patients^14^, we favor the second interpretation: the impaired light response in these patients explains the absence of the transient constriction at the onset of the disks.

In summary, the present results show that pupil size is sensitive to the interaction between light and attention (i.e. whether we pay attention to a bright or dark stimulus). This effect is robust and independent of the pretectal circuit for the pupillary light response. This provides further evidence that several components of the pupillary response can be isolated and highlights the existence of one related to the focus of covert attention.

## Electronic supplementary material

Supplementary Methods and Results
