# Supplementary material for: Pupil response components: attention-light interaction in patients with Parinaud’s syndrome
Source: Sci Rep. 2017 Aug 31;7:10283. doi: 10.1038/s41598-017-10816-x (PMC5579308; doi:10.1038/s41598-017-10816-x)
Supplement: Supplementary file 1 — Supplementary Methods and Results [file 41598_2017_10816_MOESM1_ESM.doc]

# Supplementary material for:

# Pupil response components: attention-light interaction in patients with Parinaud's syndrome

Paola Binda^1,2^, Torsten Straßer^3^, Krunoslav Stingl^3^, Paul Richter^3^, Tobias Peters^3^, Helmut Wilhelm^3^, Barbara Wilhelm^3^, Carina Kelbsch^3^

# Supplementary methods

### Equipment

Experiments took place in a dark and quiet room. Subjects sat in front of a LCD monitor screen (Eizo Flexscan S2433w, resolution 1920×1200), covering 56×48 deg at the distance of 48 cm, which was maintained constant by means of a head and chin rest. The display area with gray (25 cd/m2) background extended over the central 30×30 deg area (delimiting the area of screen that was actually used for the experiment, and that is represented in Figure 1A); the rest of the screen was turned black to reduce overall illumination. A PC (ThinkPad T530) running PsychoPy2 controlled the presentation of the stimuli while a camera (DMK 23UV024, The Imaging Source Europe GmbH, Bremen, Germany, with 50 mm TV lens 1:1.4 and an infrared-band-pass filter) positioned below the screen, recorded the image of the participant’s right eye at 100 Hz. The eye was illuminated using an infrared LED. Images were sent to the PC, where in-house software identified the corneal reflection and the pupil using the Starburst-algorithm ^1^, and stored estimates of pupil diameter and gaze position. At the beginning of each session, the acquisition parameters were adjusted to the exact participant’s position and eye features; this calibration phase took approximately 15 minutes.

During acquisitions, inter-trial intervals were manually controlled and allowed for the participant to voice their response, which were recorded by the experimenter, and for the occasional blinks.

### Pupil and gaze tracking preprocessing

For each trial, we computed summary statistics of pupil size and gaze position samples (acquired at 100Hz), with the goal of comparing them across trials where attention was directed to the left/right, to the brighter/darker disk.

We chose to characterize each trial with the median gaze position and pupil size over the interval 2-8s from trial onset. Selecting this window allowed us to focus on the central part of the trial, where blink artifacts are minimal and the effect of attention on pupil size usually showing a slow build-up, e.^g.2,3,4^ is expected to have reached a steady state. However, we verified that the exact time window used for data averaging is not critical for the conclusions of the study, as one may appreciate by inspecting the full time-course of the effects shown in Figure 2.

Gaze position estimates from each trial were subtracted of the grand-average of gaze position across all trials of a given participant. In line with our previous studies, pupil size estimates were subtracted of the median pupil size in the 1s pre-stimulus interval of each trial, to reduce inter-trial variability.

The resulting statistics (median pupil or gaze position in the 2-8s interval of each trial) were analyzed with a linear-mixed model approach. Individual trials from all control subjects and the four patients are fit with a model comprising both the effect of experimental variables (fixed effects) and the variability across participants (random effects). The main fixed effects are: “attended side” (left and right) and “attended luminance” (dark and bright). Random effects were coded by allowing subject-by-subject variations of both the slope and intercept for each of the fixed effects. The model was fit using standard Matlab functions provided with the Statistics and Machine Learning Toolbox (R2015b, The Mathworks, inc.). Specifically, the function “fitlme(data,model)” fit the linear-mixed model to the data, yielding an object “lme” with associated method “anova” that returns F-stats and p-values for each of the fixed effect terms.

A preliminary analysis fit the full pupil size dataset with the complete model (attended side × attended luminance and all random effects), and revealed no interaction between attended luminance and attended side (F(1,444) = 2.763, p=0.097). Based on this, we pooled trials across attended sides and analyzed the main effect of attended luminance on pupil size separately in the patients and the control groups.

## Supplementary results

### Assessment of pupillary unrest

Immediately before the start of the experimental session, all participants underwent the Pupillographic Sleepiness Test, with testing conditions and units of measurement exactly as described in ^5^. The PST evaluates the level of pupillary unrest – the fluctuations of pupil size in the dark that typically emerge when the level of alertness is reduced, proposed to be linked to a noradrenergic pathway from the locus coeruleus to the Edinger–Westphal (EW) nucleus ^6^. Pupil fluctuations as measured by the lnPUI index were within the normal range, both for our control participants (mean lnPUI: 1.57, range: 0.58 – 2.40) and for the patients (mean lnPUI: 1.86, range: 1.27 – 2.16). The normal lnPUI in patients confirms that Parinaud’s syndrome, while depleting the pupillary response to light, has otherwise normal pupillary behavior ^7^.

We tested whether there is any correlation between the effect of attention on pupil size (reported in the main text and shown in Figure 2) and the measure of alertness provided by the PST that each subject took before starting the experiment, but found none (Pearson’s correlation between the average pupil difference in the attend dark-attend bright conditions in each participant and their lnPUI value: R = -0.14, p > .1). Although attention may be expected to correlate with the level of alertness, all participants performed well on the color detection task; this suggests that alertness and compliance with task instruction were high across all participants and it may explain the lack of correlation between attentional pupil modulations and the PST scores.

### Dynamic stimuli

We also tested a variation of the experiment presented in the main text in three of the patients and six of the controls (in a short experimental session immediately after completion of Experiment 1), using dynamic stimuli similar as in Mathot et al. ^8^. The methods were the same as for Experiment 1, with few exceptions. The display still consisted of two disks on the left and right of fixation, one bright the other black, but their luminance swapped repeatedly, with a frequency of 0.4Hz (each half-cycle comprising a smooth luminance change lasting 0.5s and a period of steady luminance lasting 0.75s). Trials lasted 30s and consisted of 12 full stimulus-cycles (Figure S1A; 2 blocks of 8 trials were collected for each participant). Again participants were instructed to attend the left or right stimulus (at the beginning of the block) and we checked compliance by having them report the number of color changes for the dot centered in the attended disk (0-3; performance was >90% in the controls and two of the patients; 62.5% correct in the third patient).

With this configuration, the overall luminance of the display remained always constant; however, the luminance in the attended region changed periodically. If pupil size depends on the attended luminance (as shown in Experiment 1), then a pupil size oscillation at the stimulus frequency should be clearly detectable. We tested for this using the approach introduced by Mathot et al. ^8^. We sample pupil size every half stimulus-cycle and take the ratio of each sample to the previous one (ratio larger than 1 means dilation, ratio smaller than 1 means constriction). If pupil size oscillates in phase with the stimulus, this “pupil change” ratio should move above/below 1 every half stimulus-cycle and the phase of this oscillation should be orthogonal when the attended stimulus starts out bright vs. dark.

Dashed lines in Figure S1B-C show these predictions, i.e. the “pupil change” traces for a pupil that is perfectly in-synch with the attended stimulus; filled gray and black symbols show the observed pupil change traces for a subset of trials where oscillations were in phase with the attended stimulus.

We quantified the proportion of such in-phase trials by defining two values, “L_dark” and “L_bright”, corresponding to the likelihood for the pupil to oscillate in phase with the stimulus that starts out dark or the other stimulus. The L_dark/L_bright ratio at the end of the trial categorizes it as oscillating in-phase with the attended stimulus (if the ratio is >1 and the attended stimulus started out dark, or the ratio is <1 and the attended stimulus started out bright) or not in-phase (in the complementary two cases).

In-phase trials represent the majority of trials in both the patients and the controls group (green bars in Figure S1D-E). This predominance of in-phase oscillations is statistically reliable in the control group (F(1,126) = 43.777, p<0.001). It fails to reach significance in the patients group (F(1,46) = 3.063, p=0.087) although, looking at the individual participants, the proportion of in-phase oscillation is above chance in two out of three patients.

While we acknowledge that sample size is small, and limits the possibility to interpret these results, we believe that these data constitute a proof-of-principle that Parinaud’s pupils, like controls’, can be entrained to a 0.4Hz attended luminance oscillation.

Like for Experiment 1, we verified that the results cannot be explained by gaze shifts in the direction of the attended stimulus. Horizontal gaze position did not differ significantly when attention was directed to the left or to the right (F(1,46) = 3.117, p=0.084 for the patients and F(1,109) = 0.108, p=0.743 for the controls). Moreover, selecting trials where gaze position was within 0.3 deg from screen center (42/48 for the patients and 76/128 for the controls) did not prevent pupil oscillations in phase with the attended stimulus (the proportion of in-phase trials remained significantly higher than chance for the controls, 80:20%, F(1,74) = 32.140, p<0.001, and actually improved for the patients, reaching 75:25%, F(1,22) = 6.078, p<0.05).

Incidentally, we note that attentional effects were readily detected in short experimental sessions: only 16 trials for this dynamic stimulus protocol, and 32 trials for the static stimulus presented in the main text. This highlights the fact that, albeit small, the attentional modulation of pupil size is robust and does not require prolonged testing, which is important for the possible applications of these paradigms as tools to study other aspects of vision/attention ^9^ or for BMI applications ^10^.

### Figure S1: Effect of attention on luminance oscillations


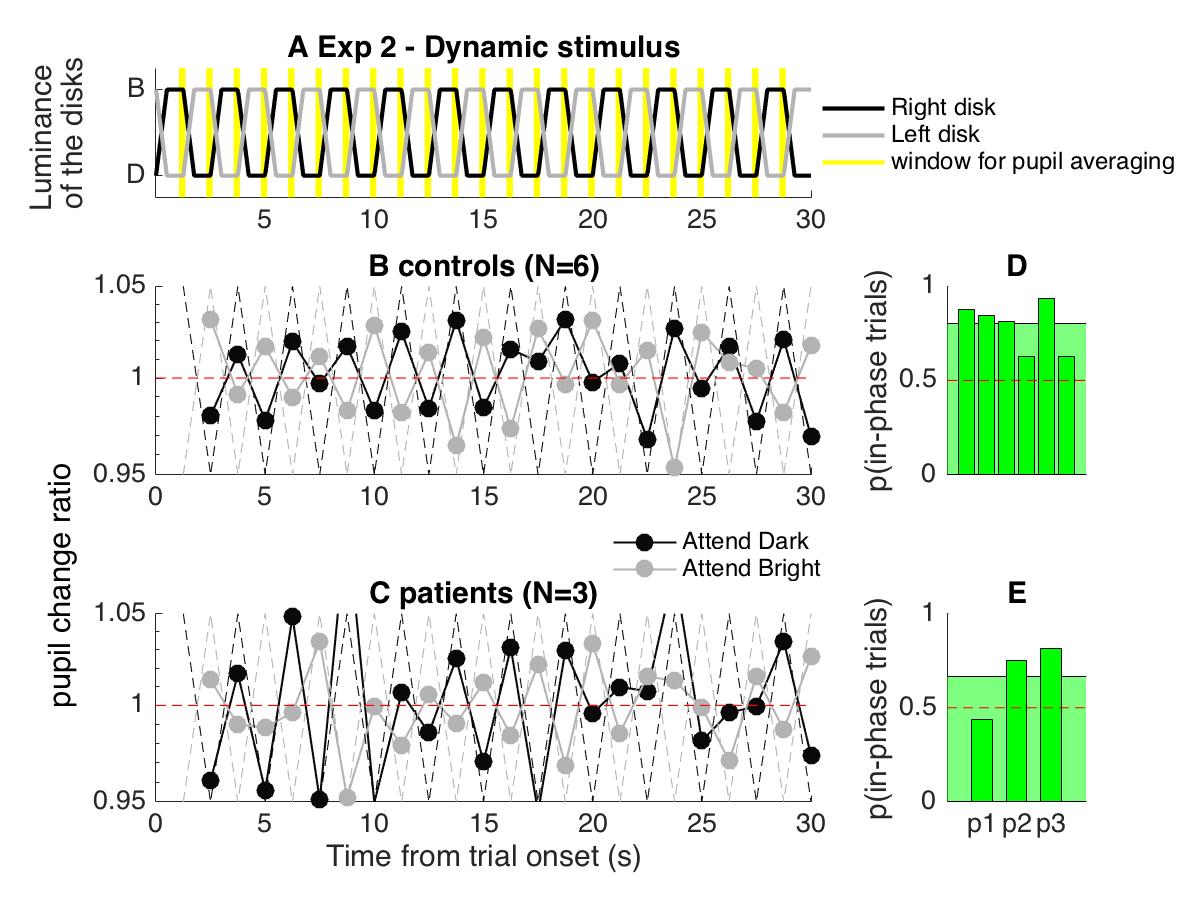


*A: time course of stimulus luminance (B and D on the y axis stand for Bright and Dark relative to the background), shown separately for the right and left disks in Figure 1A of the main text. The disks swapped positions periodically with a frequency of 0.4Hz over each 30s trial. Participants maintained their attention on one side (left or right); we define “Attend Dark” trials those where the stimulus on the attended side started out dark (like the right stimulus in the case shown here). Pupil size is sampled at the end of each stimulus half cycle, averaging over the 0.125s windows (yellow shading).*

*B-C: dashed lines predicted show the predicted pupil change ratios in the two attention conditions (“Attend Dark” and “Attend Bright” trials). Filled symbols show actual pupil change ratios, averaged across trials categorized as “in-phase” with the attended stimulus. Panels D-E give the proportion of such in-phase trials, for each participant and averaged across subjects (large background bar). The red dashed line shows the proportion of in-phase trial expected by chance (0.5).*

## Supplementary References

1 Li, D. & Parkhurst, D. J. Starburst : A robust algorithm for video-based eye tracking. *Image (Rochester, N.Y.)* (2005).

2 Binda, P., Pereverzeva, M. & Murray, S. O. Attention to bright surfaces enhances the pupillary light reflex. *J Neurosci* **33**, 2199-2204, doi:10.1523/JNEUROSCI.3440-12.2013 (2013).

3 Binda, P., Pereverzeva, M. & Murray, S. O. Pupil size reflects the focus of feature-based attention. *J Neurophysiol* **112**, 3046-3052, doi:10.1152/jn.00502.2014 (2014).

4 Binda, P. & Murray, S. O. Spatial attention increases the pupillary response to light changes. *J Vis* **15**, 1, doi:10.1167/15.2.1 (2015).

5 Wilhelm, B. *et al.* Short-term reproducibility and variability of the pupillographic sleepiness test. *American journal of human biology : the official journal of the Human Biology Council* **27**, 862-866, doi:10.1002/ajhb.22726 (2015).

6 Koss, M. C., Gherezghiher, T. & Nomura, A. CNS adrenergic inhibition of parasympathetic oculomotor tone. *J Auton Nerv Syst* **10**, 55-68 (1984).

7 Wilhelm, B. J., Wilhelm, H., Moro, S. & Barbur, J. L. Pupil response components: studies in patients with Parinaud's syndrome. *Brain* **125**, 2296-2307 (2002).

8 Mathot, S., Melmi, J. B., van der Linden, L. & Van der Stigchel, S. The Mind-Writing Pupil: A Human-Computer Interface Based on Decoding of Covert Attention through Pupillometry. *PLoS One* **11**, e0148805, doi:10.1371/journal.pone.0148805 (2016).

9 Binda, P. & Murray, S. O. Keeping a large-pupilled eye on high-level visual processing. *Trends Cogn Sci* **19**, 1-3, doi:10.1016/j.tics.2014.11.002 (2015).

10 Mathot, S. & Van der Stigchel, S. New Light on the Mind's Eye: The Pupillary Light Response as Active Vision. *Current directions in psychological science* **24**, 374-378, doi:10.1177/0963721415593725 (2015).
